# Genetic diversity and population structure of the natural population of *Helicoverpa armigera* in Northwest China using Genotyping by Sequencing (GBS) technology

**DOI:** 10.1371/journal.pone.0336253

**Published:** 2025-11-06

**Authors:** Zhongxiong Ning, Jiali He, Jianhao Dong, Weizheng Li, Ruihao Li, Zhengzhen Gao, Dongmei Wang, Haiqiang Li, Bing Liu, Yujiao Wang, Wei Lu

**Affiliations:** 1 Engineering Research Centre of Cotton, Ministry of Education, College of Agriculture, Xinjiang Agricultural University, Urumqi, China; 2 Key Laboratory of Integrated Pest Management on Crop in Northwestern Oasis, Ministry of Agriculture and Rural Affairs, Institute of Plant Protection, Xinjiang Academy of Agricultural Sciences, Urumqi, China; 3 State Key Laboratory for Biology of Plant Diseases and Insect Pests, Institute of Plant Protection, Chinese Academy of Agricultural Sciences, Beijing, China; 4 Western Agricultural Research Center, Chinese Academy of Agricultural Sciences, Changji, China; PMAS Arid Agriculture University: PMAS-Arid Agriculture University Rawalpindi, PAKISTAN

## Abstract

Characterizing the genetic diversity and population structure can determine whether there is gene flow of the natural population of *Helicoverpa armigera* (Hübner) under disparate climate and habitat conditions in Northwest China. In this paper, *H. armigera* was genotyped in various regions of Xinjiang using Genotyping-by-Sequencing (GBS). The samples were compared using the single nucleotide polymorphism (SNP) and insertion deletion (InDel) marker data. The SNPs were used to analyze the population structure and five subgroups were obtained, which was further confirmed by principal component analysis (PCA). The phylogenetic tree identified five cluster populations of *H. armigera*. The average values of polymorphic information content (PIC) and genetic differentiation index (Fst) are 0.1783 and 0.1293, respectively, which are at a high level. The phylogenetic tree differentiation also indicates that the genetic diversity of cotton bollworm populations in different regions of Xinjiang is low diversity, moderate differentiation, and widespread gene flow. According to correlation analysis of the source of feeding on host plants (Bt cotton and non Bt crops) of H. armigera, seven SNPs with significant differences were obtained. The most significant SNP sequence was compared with the whole genome of *H. armigera*, and 10 candidate genes were screened. Whether the candidate genes function are related to Bt resistance needs further verification. This study can provide scientific basis for screening Bt resistance genes and formulating refuge strategy of *H. armigera* in Northwest China.

## Introduction

Xinjiang Uygur Autonomous Region (called Xinjiang) is located in the northwest of China, which is an important high-quality cotton production base and is the largest cotton-producing region and commercial cotton base in China [1,2]. According to the data of the Statistical Yearbook, in 2020, Xinjiang’s cotton planted area was approximately 1,864,000 hectares with a yield of 5.91 million tons, accounting for 58.82% of the national planted area and 87.33% of the national total output. The *H. armigera* belongs to the family Noctuidae of the order Lepidoptera [2,3], and this species has the characteristics of high omnivory, long-distance migration ability, facultative diapause and strong reproduction, which enables the *H. armigera* to adapt to various ecological environments [4–7]. In Xinjiang, *H. armigera*s are hosted by many plants in addition to cotton and also harm such crops as corn, wheat, tomato, sunflower, and peas [8]. When bollworms damage cotton, they mainly harm young tips, buds, flowers and bolls. The second and third generations of *H. armigera* are the most serious pests, exhibiting population densities of up to 38 per 100 plants, insect strain rates of 18 ~ 26%, and crop yields of 20 ~ 30% [9,10].

Different from the cotton region of the North China Plain, Xinjiang is characterized by “two basins lying between three mountain ranges” (the Altay Mountains, the Jungle Basin, the Tianshan Mountains, the Tarim Basin, and the Kunlun Mountains in order from north to south), the climate conditions are quite different in Southern and Northern Xinjiang. [11–14]. The main planting areas are mainly concentrated in oasis areas, which have rich water systems at the edge of the basin; therefore, these planting areas exhibit distribution patterns of isolated islands or beads [1,15]. Moreover, the cotton planting structure in Xinjiang is relatively simple, as Bt cotton accounts for 85% of cotton planted, and the natural refuge area is insufficient. The natural population of *H. armigera* may develop resistance to Bt under the long-term selection pressure of Bt cotton [10,16–22]. Are mountain ranges and basin deserts geographically blocking the migration of bollworms? Is there gene flow among natural populations of *H. armigera* in the eastern, southern and northern regions of Xinjiang? How genetically diverse are the bollworms in this region? At present, there are few answers to these questions.

Genotyping-by-sequencing (GBS) is a common simplified genome sequencing technology that is based on the principle of enzymatic digestion of genomic DNA followed by high-throughput sequencing of the sequences at both ends of the enzymatic digestion fragment [23,24]. GBS technology can flexibly adjust the tags needed to capture the restriction sites according to the research purpose to control the range of the capture sequence [23]. GBS has been widely used in the development of high-density and high-precision SNP molecular markers for crops [24,25], but there are few studies on the genetic diversity of agricultural pests. Therefore, this study on Xinjiang’s eight major cotton-producing areas, namely, Aksu (AKS), Alaer (ALR), Changji (CJ), Hami (HM), Korla (KEL), Kashgar (KS), Shache (SC), and Shawan (SW), collected natural populations of individual *H. armigera*, used GBS simplified genome sequencing technology to sequence their genomes, built the GBS library, performed specific-locus amplified fragment sequencing, and developed a large number of single nucleotide polymorphisms (SNPs) in *H. armigera* genome tags. Based on the screening of SNP marker, the genetic diversity and population structure of *H. armigera* in various regions of Xinjiang were analyzed. The eastern, southern, and northern populations of *H. armigera* in Xinjiang were clearly identified due to genetic flow among natural populations. According to the source of bollworm feeding on host plants (Bt cotton and non Bt crops), the association analysis was carried out to screen out some SNP sites with significant differences and whether they are candidate genes related to Bt resistance. The results of this study helped to elucidate which refuge strategy formulation was suitable, as well as helping to provide a scientific basis for Bt resistance monitoring and management.

## Materials and methods

### Sampling genomes

Forty-nine samples of *H. armigera* were obtained from traps in Xinjiang Aksu and the latitude and longitude are 40.523456 ° N and 80.321789 ° E (ten samples), Alaer and the latitude and longitude are 40.637896 ° N and 81.234567 ° E (five samples), Changji and the latitude and longitude are 44.003062 ° N and 87.32926 ° E (five samples), Hami and the latitude and longitude are 42.756123 ° N and 93.481357 ° E (five samples), the latitude and longitude of the sampled cotton fields in Korla are 41.682345 ° N and 86.274561 ° E (five samples), the latitude and longitude of the cotton fields sampled in Kashgar are 39.512345 ° N and 76.123456 ° E (five samples), the latitude and longitude of the cotton fields sampled in Shache are 38.576543 ° N and 77.312345 ° E (five samples), and the latitude and longitude of the cotton field sampled in Shawan are 44.212345 ° N and 85.654321 ° E (four samples). These sampled cotton fields are all planted with Bt cotton, separated into 2 mL centrifuge tubes, and stored at −20°C.

Using a Cell/Tissue Genomic DNA Extraction Kit from Hunchek Biological Technology Co., Ltd. (Beijing), genomic DNA was extracted from forty-nine *H. armigera*, and 1% agarose gel electrophoresis was utilized to determine the integrity of DNA, while a NanoDrop 1000C spectrophotometer (American NanoDrop, ND-1000) and Qubit 3.0 fluorometer were employed to measure the concentration and purity of the DNA samples to ensure that their quality satisfied the requirements of sequencing DNA (namely, an OD260/OD280 ratio distribution between 1.8-2.2). The concentration of the DNA was determined to be> 50 ng/μL, and total of > 2 g DNA was obtained. Samples were stored at -20°C for later use.

### Enzyme digestion to build library

Genomic DNA of forty-nine *H. armigera* samples was sequenced using genotyping by sequencing (GBS) technology. The restriction endonucleases Mse I and Sac I were used to fully digest the whole genomes of the samples. Fragments measuring 300−400 bp were retrieved to construct the GBS library. The library was sequenced by the Paired-End 150-bp (PE150) Illumina Nova Seq sequencing platform and completed by GenoSecc Genomics Co., Ltd. The *H. armigera* reference genome address is Chr13: 2,663K-3,937K - Genome Data Viewer - NCBI.

### Label SNP and InDel analysis

The original data obtained by sequencing were transformed into original sequencing sequences, or reads, by base calling analysis, and the reads were filtered according to the following principles: reads with ≥10% unknown nucleotides were filtered, reads consisting of >30% low-quality nucleotides were filtered, and reads that were of low quality at the 3’end were filtered. High-quality double-ended clean reads were obtained, and sequencing quality and data volume were evaluated. The comparison rate and base mass content were evaluated by comparing data to judge the validity and accuracy of the experimental process. Clean sequencing reads were compared to the reference genome using BWA software (version 0.7.15-R1140) [26], and mutant SNPs and InDels (insert missing marker) were analyzed using GATK software (version 3.7) [27] to obtain marker data.

### Population genetic structure and genetic relationship analysis

The developed SNPs and InDel markers were filtered according to the criteria of completeness >0.8 and secondary allele frequency (MAF) >0.05 [28]. Based on the screened population SNPs with high consistency, the statistical software tools Structure (version 2.3.4), Plink (version v1.90p) and Mega7 (version 7.0) were used to analyze the population evolutionary tree and population structure [29–31]. Stuck software (version 1.48) [32] was used to calculate the expected heterozygosity (HE), observed heterozygosity (HO), number of inbred lines (Fis) and genetic differentiation index (Fst). Two genetic diversity indexes, the Shannon index (H) and polymorphic information content (PIC), were also calculated.

### Phylogenetic tree analysis

A phylogenetic tree is a branch diagram or tree that describes the order of differentiation between populations and is used to represent the evolutionary relationship between populations. According to the PCA scatter plot, neighbor-joining methods in Mega 7 (version 7.0) software were used to construct the phylogenetic tree, and ggtree (version 1.7.10) was used for visualization.

### Linkage imbalance analysis and genome-wide association analysis

In this study, VCF Tools software (version 0.1.17) was utilized to calculate the r2 value between two loci, and was employed to estimate the LD degree in the population. Gemma (version 0.98.1) software was used to perform genome-wide association analysis using a mixed linear model with the addition of genetic relationships as a random effect. GEC (version 0.2) software developed by Li *et al*., [33] was utilized to calculate the number of effective markers in the population, and its suggested value (1/n, where n is the number of effective markers) was employed as the threshold for association analysis.

## Results

### Database construction evaluation and sequencing quality evaluation

The size of the cotton-bollworm control genome in this study was 374.30 Mb, and the comparison of the control sequencing reads with the reference genome was conducted through the MEM algorithm of BWA software (version 0.7.15-R1140). The results demonstrated that the double-ended comparison efficiency of the experiment was 77.47%, which was largely normal, indicating that the library was constructed normally and without contamination. The sequencing quality value (Q) is an important index for evaluating the error rate of single-base sequencing. The higher the Q value is, the lower the error rate of single-base sequencing is. In this study, a total of 289.383 million reads were obtained from forty-nine *H. armigera* samples. The average Q30 (the probability of a base sequencing error is 0.001; therefore, the base’s Q30 was 94.30%, and the average GC content was 43.77%. Q30 data showed that the sequencing quality was high and that the sequencing results were reliable.

### SNP and InDel marker information statistics

**Fig 1:**
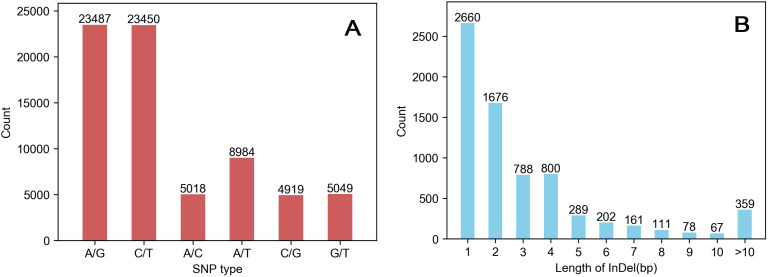
Statistical analysis of SNP variation types (A). InDel (insert missing mark) length statistics (B).

Reads generated by sequencing were compared to reference sequences obtained using BWA software, and mutations were detected together with samples using GATK (version 3.7) software, including SNPs and InDels. A total of 78,098 mutation loci were obtained in this study. The average genome coverage of all samples was 5.34%, and the average coverage depth was 23.78%. Among these mutations, 70,907 were SNPs, and 7,191 were InDels. Among the SNPs, 46,937 were conversion-type (A/G and C/T), while 23,970 were transversion-type (A/C, A/T, C/G and G/T), and the conversion transversion ratio (TS/TV) was 1.96. (**Fig 1**)

### Population structure analysis

#### Structure analysis.

**Fig 2:**
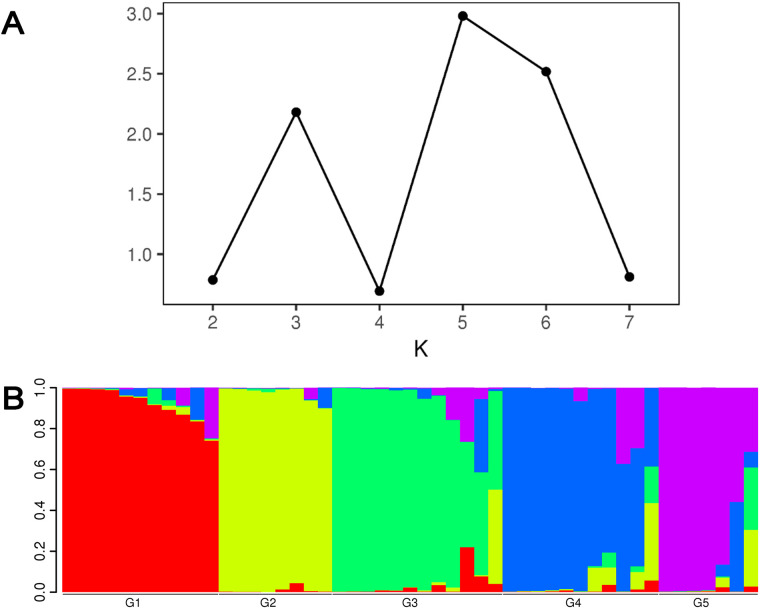
Genetic population number of *H.* *armigera* (K = 1-7) in various regions in Xinjiang was evaluated by Structure software (A). Population structure of *H. armigera* in various regions in Xinjiang was analyzed based on SNPs (B).

**Fig 3:**
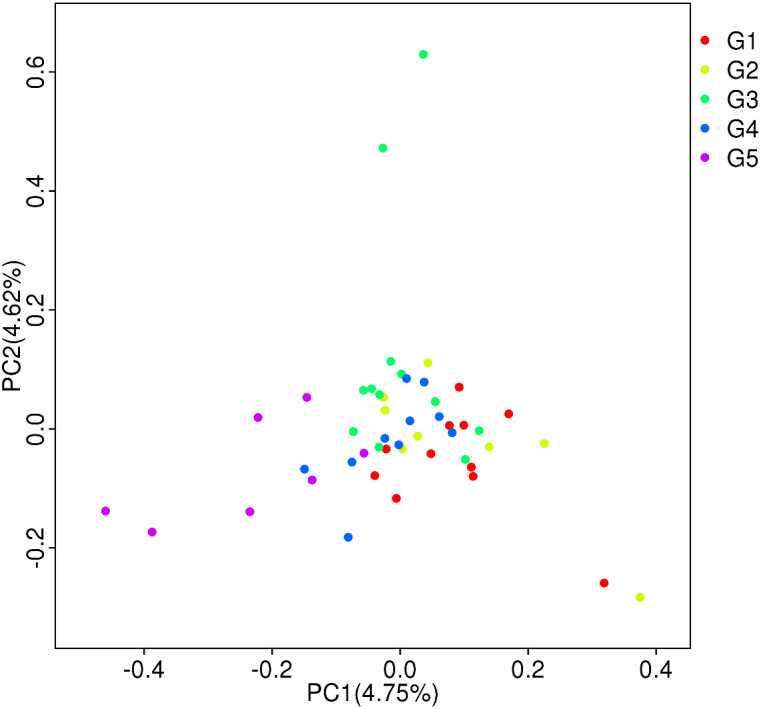
Principal component analysis of the natural population of cotton bollworm in various regions of Xinjiang.

Based on filtered SNPs, the population structure of forty-nine *H. armigera* samples was analyzed by Structure (Version 2.3.4) software (**Fig 2**). The results show that when K = 5, ΔK peaks, indicating that the optimal genetic group number is five. The PCA results (**Fig 3**) were consistent with the results of the structure analysis. The experimental materials could be divided into five subgroups. Among these subgroups, there were eleven samples from subgroup G1, primarily from Changji and Shache. There were eight samples in subgroup G2, primarily from Aksu. There were twelve samples from subgroup G3, primarily from Kashgar. There were eleven samples from subgroup G4, primarily from Changji. Seven samples were collected from subgroup G5, primarily from Shawan. The distribution frequencies of the five subgroups were 22.45%, 16.33%, 24.49%, 22.45% and 14.28%, respectively. In PCA clustering analysis, the genetic backgrounds of *H. armigera* samples from various regions of the 5 subpopulations were mostly similar, but there were individuals with very different genetic backgrounds in all five subpopulations, that is, G1-G5.

#### Phylogenetic tree analysis.

**Fig 4:**
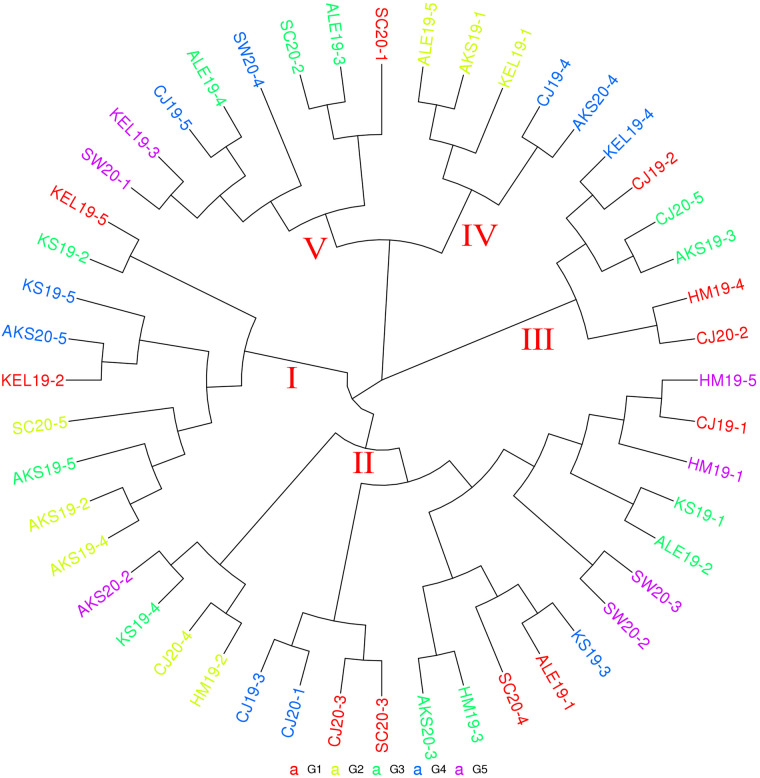
Phylogenetic tree of the natural population of *H. armigera* in various regions of Xinjiang Note: Regarding the numbers after the abbreviations for place names, 19 means the sample was taken in 2019, 20 means the sample was taken in 2020, and “-1, -2, -3...” represents the sample number.

The phylogenetic tree results are shown in Fig 4. (**Fig 4**) The results of phylogenetic tree analysis and cluster analysis were the same and could be roughly divided into five different clusters (I, II, III, IV, and V).

### Genetic diversity analysis and differentiation of *H. armigera* populations

#### Genetic diversity analysis of *H. armigera.*

The observed heterozygosity and expected heterozygosity of various *H. armigera* populations in Xinjiang ranged from 0.1974 to 0.2177 and from 0.1076 to 0.1810 with averages of 0.2089 and 0.1199, respectively. The observed heterozygosity was the lowest in the CJ19 population and the highest in the AKS20 population, while the expected heterozygosity was the lowest in the SW20 population and the highest in the ALE19 population.

The inbreeding number Fis in various *H. armigera* populations in Xinjiang ranged from 0.137 to 0.2842 with an average of 0.2514. The inbreeding number of the populations used in various populations was greater than 0, among which nine populations were greater than 0.2. AKS20 has the smallest Fis, while the other nine populations have more normal 0 values when Fis values deviate from equilibrium; in other words, the observed heterozygosity and the expected heterozygosity are significantly different, and the population has seriously deviated from the normal random mating mode. The Fis value of AKS20 is the closest to 0, indicating that the observed heterozygosity and the expected heterozygosity of this population are less different, and the population is close to the ideal state of free mating.

The polymorphism information content (PIC) of the 10 populations varied from 0.1721 to 0.1821 with an average value of 0.1783. The polymorphism information content (PIC) of the 10 populations varied from 0 to 0.25, and the diversity of the 10 populations was low and abundant (as shown in Table 1).

**Table 1:** Genetic diversity index of natural populations of *H. armigera* in various regions of Xinjiang.

Pop ID	He	Ho	Fis	H	PIC
AKS19	0.2153	0.1110	0.2842	0.3372	0.1812
AKS20	0.2120	0.1810	0.1370	0.3340	0.1794
ALE19	0.2177	0.1302	0.2506	0.3391	0.1821
CJ19	0.2047	0.1076	0.2677	0.3307	0.1778
CJ20	0.2049	0.1124	0.2586	0.3320	0.1785
HM19	0.2082	0.1085	0.2734	0.3300	0.1773
KEL19	0.2089	0.1091	0.2740	0.3313	0.1781
KS19	0.2092	0.1083	0.2757	0.3301	0.1775
SC20	0.2102	0.1120	0.2707	0.3331	0.1791
SW20	0.1974	0.1192	0.2218	0.3189	0.1721

### Genetic differentiation among *H. armigera*

Stacks software was utilized to calculate the genetic differentiation index among bollworms. Paired Fst values between 10 bollworm populations ranged from 0.1156 to 0.1486 (as shown in **Table 2**) with an average Fst of 0.1299. Among the 90 paired populations, the Fst values of all paired populations were less than 0.15. These results indicated that the genetic differentiation of most populations of *H. armigera* was moderate, which indicated that there was extensive gene exchange among natural populations of *H. armigera* in Xinjiang.

**Table 2:** Genetic differentiation coefficient (FST value) among natural populations of *H. armigera* in various regions of Xinjiang.

ID	AKS19	AKS20	ALE19	CJ19	CJ20	HM19	KEL19	KS19	SC20	SW20
AKS19	0									
AKS20	0.1187	0								
ALE19	0.1192	0.1156	0							
CJ19	0.1297	0.1261	0.1250	0						
CJ20	0.1283	0.1256	0.1250	0.1350	0					
HM19	0.1253	0.1221	0.1214	0.1319	0.1308	0				
KEL19	0.1254	0.1225	0.1219	0.1331	0.1320	0.1286	0			
KS19	0.1248	0.1220	0.1208	0.1319	0.1307	0.1269	0.1286	0		
SC20	0.1262	0.1231	0.1188	0.1330	0.1310	0.1292	0.1306	0.1289	0	
SW20	0.1421	0.1399	0.1369	0.1486	0.1483	0.1447	0.1456	0.1452	0.1461	0

#### Chain disequilibrium analysis.

**Fig 5:**
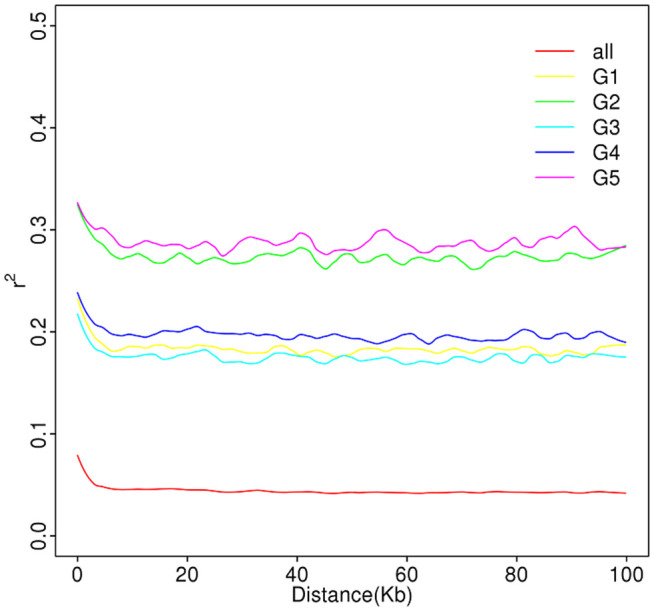
LD values of five *H. armigera* subpopulations in various regions of Xinjiang.

Using a statistical method to calculate the LD decline level of five subpopulations of *H. armigera* in eight regions in Xinjiang (**Fig 5**), the R2 value of G5 was the largest, and the R2 value of G3 was the smallest. The LD coefficients of the four subgroups could be rankedas G5 > G2 > G4 > G1 > G3, and the LD attenuation curves of the five subgroups were nearly parallel to the X axis. When measuring the linkage imbalance degree r2 was less than 0.33, which indicated that the LD of the population was mainly caused by recombination, while mutation had little effect on LD. Linkage disequilibrium exists in all collinearity, which indicates that the linkage groups have undergone historical reorganization. This finding may be observed because the markers among different linkage groups are independent, and the linkage imbalance among these markers is easily broken, while the markers in the same linkage group have linkage relationships; therefore, the linkage imbalance degree is higher, the similarity degree between individuals is higher, and the genetic diversity is lower. There was no significant attenuation of LD in the five subgroups, and the degree of linkage imbalance was very high.

#### Genome-wide association analysis.

**Fig 6:**
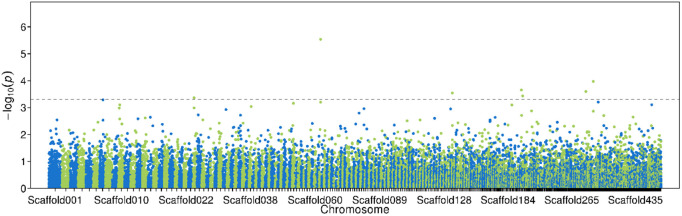
Manhatan diagram for correlation analysis of feeding traits of *H. armigera.* The dotted line represents the threshold of potential genome-wide significant association: -log10 = 3.34.

UPLC/MS/MS was used to determine the content of gossypol (a special compound of cotton) in the body of *H. armigera* [34]. It can be known that *H. armigera* comes from Bt cotton or non Bt crops. The Manhattan diagram drawn by Gemma (version 0.98.1) based on the host plant sources (Bt cotton and non-Bt crops) on which *H. armigera* feeds is shown below. The number of effective markers calculated by GEC software was 7142, and the corresponding threshold of association analysis was P = 0.000145, which was 3.34 after -log10 conversion. Genome-wide association analysis was conducted on various regions of *H. armigera* samples, and the results are shown in Fig 6 (**Fig 6**).

Compared with the genome of *H. armigera*, the results were as follows there is an associated SNP on scaffold062, scaffold134, scaffold198, scaffold200, scaffold290 and scaffold304, among which the SNP on scaffold062 is the most significant, and the accession number of its source sequence was NW_018395451.1, position 734791. Ten candidate genes were screened from 600000–800000 upstream and downstream of the locus (as shown in Table 3).

**Table 3:** Information of genes associated with SNP source sequences of *H. armigera* (comparing the genome of *H. armigera*).

Accession	Start	Stop	Strand	GeneID	Locus	Locus tag	Protein product	Length	Protein Name
NW_018395451.1	637748	641049	+	110373725	LOC110373725	–	XP_021186740.1	171	uncharacterized protein LOC110373725
NW_018395451.1	645275	700297	–	110373727	LOC110373727	–	XP_021186744.1	861	protein trachealess isoform X2
NW_018395451.1	645275	700297	–	110373727	LOC110373727	–	XP_021186743.1	879	protein trachealess isoform X1
NW_018395451.1	843567	844328	+	110373738	LOC110373738	–	XP_021186761.1	253	uncharacterized protein LOC110373738
NW_018395451.1	850218	870092	–	110373737	LOC110373737	–	XP_021186759.1	1040	tubulin polyglutamylase TTLL4-like isoform X4
NW_018395451.1	850218	870092	–	110373737	LOC110373737	–	XP_021186760.1	1066	uncharacterized protein LOC110373737 isoform X5
NW_018395451.1	850218	870092	–	110373737	LOC110373737	–	XP_021186758.1	1111	uncharacterized protein LOC110373737 isoform X3
NW_018395451.1	850218	870092	–	110373737	LOC110373737	–	XP_021186756.1	1131	uncharacterized protein LOC110373737 isoform X1
NW_018395451.1	850218	870092	–	110373737	LOC110373737	–	XP_021186757.1	1118	uncharacterized protein LOC110373737 isoform X2
NW_018395451.1	872095	873249	+	110373723	LOC110373723	–	XP_021186738.1	384	uncharacterized protein LOC110373723

## Discussion

Construction of genetic maps based on GBS simplified genome technology can cover the entire genome, eliminate limits for reference genomes, and reduce the complexity of the genome [4,25]. With this technology, the data volume is low, operation is easy, and costs are low, and this method is therefore widely applied in the development of high-density, high-precision crop SNP marker research [35]. At present, GBS plays an important role in the study of the genetic diversity and population resources of agricultural pests [36]. Wosula *et al*. genotyped Bemisiatabaci from cassava from 8 countries in Africa and identified 6 major groups, which were further confirmed by PCA and multidimensional analysis, with significant gene exchange being detected among the groups [37]. Perepa *et al*. used 85 single nucleotide polymorphisms (SNPs) to genotype populations of Helicoverpa zea captured from Landisville and Rock Springs, Pennsylvania, USA, in 2002, 2005, 2016, and 2018. Genotypic samples were divided into 16 putative populations according to collection time and location. High genetic diversity within populations and low genetic differentiation between populations were observed in 2002 and 2005 [38].

Studies based on DNA genes and limited nucleotide markers have shown that the population structure of *H. armigera* generally lacks subgroup structure, even among populations that are geographically far apart [39–42]. Anderson *et al*. used genome-wide SNP markers to demonstrate differences between Australian/New Zealand bollworm subspecies and Old World bollworm subspecies but not among global populations [43]. Genotypes of *H. armigera* in southeastern South America and northern/central Brazil and southwestern *H. armigera* populations are rare and/or lack the dominant mtDNA haploid phenomenon; thus, *H. armigera* invasion may employ two different paths in Brazil and may be associated with the import and export of agricultural products and horticultural products [44].

Xinjiang Province covers an area of approximately 1/6 of China’s land area and is distinguished from the cotton region of the North China Plain by its unique geomorphological features of “three mountain ranges two basins” [11–14]. Unfortunately, in 2020, COVID-19 virus was rampaging around the world. Due to epidemic prevention requirements and control and travel restrictions, at some sample collection sites in Xinjiang, *H. armigera* samples were not collected in 2020. Among the population structures characterized in this study, the genetic diversity of the G3 subgroup was the most complex, and the samples included 6 different regions. The G1 subgroup is mainly from Changji and Shachi, but the distance between the two regions is more than 1400 km, meaning that the bollworms need to bypass the Tianshan Mountains and the Taklimakan Desert to migrate. A reasonable theory is that this spread is probably the result of natural migration. The migration of *H. armigera* in China usually occurs between insect overwintering and non-overwintering areas, and it usually takes 1–2 nights to migrate 200–500 km to northern non-overwintering areas [45,46]. A second theory is related to the fact that Kashgar is a special economic zone in western China. Under the influence of economic trade, agricultural products with *H. armigera* eggs circulate between southern and northern Xinjiang. Alternatively, this spread may be related to different pest control/management strategies. Detailed conclusions need to be proven by means of radar monitoring, morphological changes, feeding biology, flight biology and reproductive biology [46]. The genetic diversity of the G2 subgroup is relatively conservative, and the intercropping area of jujube, walnut, apple and cotton is relatively large in Aksu. It may be surmised that bollworms, as omnivorous pests, can choose among more host plants or may be related to the local characteristic fruit-cotton intercropping pattern. The G4 and G5 subpopulations were obtained from Changji and Shawan, respectively, which are in northern Xinjiang and less than 200 km apart. The genetic structures of the samples were mixed, suggesting that there might be frequent gene exchange between natural populations of H. armigera in these two locations.

In 2018, Wang Dongmei, Yang Xianming, and others used the cytochrome c oxidase subunit I (COI) gene fragment to study the population structure of cotton bollworm in Xinjiang, China. The final comparison showed 28 haplotypes, with 23 in southern Xinjiang, 5 in eastern Xinjiang, and 13 in northern Xinjiang. The average genetic differentiation index measured in all samples was 0.1321, which is at a high level, indicating that the genetic diversity of the cotton bollworm population is low diversity, moderate differentiation, and extensive gene flow [8]. After 2025, Hou, Bofeng et al. analyzed the population structure of cotton bollworms in different regions of Xinjiang. The population structure analysis showed significant gene flow in various areas of Xinjiang, but the differences between the westernmost collection sites of Kashgar and Shawan, as well as with other collection sites, were the greatest. The cotton bollworms in Shawan and Kashgar had genetic differences among each other and with other populations in Xinjiang at the whole genome level [47].

In this study, the natural population of *H. armigera* in Xinjiang was sequenced with the aim of developing SNPs that may be associated with Bt resistance by analyzing the genotypes of SNPs and the host plant sources (Bt cotton and non-Bt crops) that the bollworm feeds on. Due to the relatively small study sample size (n = 49), the reported Tassel analysis process for large samples [48] was not employed in the construction of the simplified genome reference sequence, and the stack analysis process [32], which is more suitable for small groups, was applied. Ten candidate genes were screened by comparing the most significant SNPs, and their functions need to be further studied.

## Conclusions

The library constructed by GSB technology is pollution-free, high-quality, and the sequencing results are reliable; a large number of SNPs have been successfully screened at the whole genome level of *H. armigera*. The natural populations of *H. armigera* in Xinjiang were divided into five subgroups and five cluster groups by population structure analysis and cluster analysis, and the results were consistent. The low genetic diversity of *H. armigera* populations in eight regions indicated that there was extensive gene flow among them. Due to the large planting area and single planting mode of Bt cotton in Xinjiang, *H. armigera* has potential risk of BT resistance under the long-term selection pressure of Bt cotton. Therefore, it may be necessary to consider the whole Xinjiang Province as a spatial scale when formulating refuge strategies in the future. Seven significant SNP loci and ten candidate genes were obtained by association analysis, and their gene functions need to be further studied in subsequent experiments.

## Supporting information

S1 Table Table S1 Statistical table of sequencing data. (DOCX)

S2 Table Comparison rate of sequencing data. (DOCX)

S3 Table Statistical table of cotton bollworm genome coverage and coverage depth. (DOCX)

S4 Table ANNOVAR annotation classification. (DOCX)

S5 Table Summary of SNP annotation results. (DOCX)

S6 Table Summary of InDel annotation results. (DOCX)

S7 Table Statistical table of variation quantity and density of SNP and InDel markers. (DOCX)
