# Supplementary material for: Genetic diversity and population structure of the natural population of Helicoverpa armigera in Northwest China using Genotyping by Sequencing (GBS) technology
Source: PLoS One. 2025 Nov 6;20(11):e0336253. doi: 10.1371/journal.pone.0336253 (PMC12591424; doi:10.1371/journal.pone.0336253)
Supplement: S1 Table — (DOCX) [file pone.0336253.s001.docx]

**Table S1 Statistical table of sequencing data**

| Sample | Reads | Bases | GC(%) | Q20(%) | Q30(%) |
| --- | --- | --- | --- | --- | --- |
| AKS19-1 | 2907723 | 736615373 | 42.72 | 98.44 | 94.60 |
| AKS19-2 | 2897811 | 733809109 | 42.50 | 98.11 | 93.38 |
| AKS19-3 | 3078904 | 786555071 | 44.79 | 98.43 | 94.56 |
| AKS19-4 | 2396320 | 602245564 | 42.25 | 98.16 | 93.50 |
| AKS19-5 | 3162823 | 825396718 | 44.84 | 98.28 | 93.81 |
| AKS20-1 | 2010151 | 531419561 | 53.28 | 98.53 | 94.48 |
| AKS20-2 | 3557576 | 906422355 | 42.02 | 98.29 | 94.00 |
| AKS20-3 | 2035939 | 535866388 | 45.92 | 98.36 | 94.16 |
| AKS20-4 | 3288536 | 832442561 | 41.96 | 98.27 | 94.05 |
| AKS20-5 | 2678466 | 699673843 | 47.30 | 98.37 | 94.17 |
| ALE19-1 | 3474584 | 859453641 | 42.81 | 98.37 | 94.51 |
| ALE19-2 | 3164167 | 800034400 | 43.48 | 98.11 | 93.42 |
| ALE19-3 | 2796803 | 734928239 | 49.17 | 98.20 | 93.47 |
| ALE19-4 | 3065895 | 788446939 | 44.50 | 98.38 | 94.41 |
| ALE19-5 | 2586490 | 684418862 | 43.31 | 98.39 | 94.28 |
| CJ19-1 | 2710358 | 653720666 | 42.18 | 98.57 | 95.20 |
| CJ19-2 | 2785730 | 657101434 | 42.28 | 98.32 | 94.65 |
| CJ19-3 | 3162006 | 752208765 | 42.08 | 98.38 | 94.70 |
| CJ19-4 | 2125493 | 557088542 | 42.12 | 98.53 | 95.02 |
| CJ19-5 | 2219606 | 537795873 | 41.93 | 98.57 | 95.26 |
| CJ20-1 | 1552089 | 364274701 | 41.63 | 98.84 | 96.28 |
| CJ20-2 | 3150473 | 760773071 | 43.03 | 98.44 | 94.76 |
| CJ20-3 | 2968838 | 715533398 | 43.05 | 98.25 | 94.19 |
| CJ20-4 | 2244501 | 557902259 | 42.93 | 98.32 | 94.20 |
| CJ20-5 | 2612318 | 663054395 | 41.27 | 98.43 | 94.68 |
| HM19-1 | 2964083 | 774168994 | 44.13 | 98.39 | 94.33 |
| HM19-2 | 3698193 | 929244092 | 44.33 | 98.39 | 94.43 |
| HM19-3 | 3430109 | 825843532 | 43.28 | 98.23 | 94.25 |
| HM19-4 | 2387831 | 609803620 | 42.68 | 98.38 | 94.50 |
| HM19-5 | 3227345 | 824399930 | 44.10 | 98.36 | 94.35 |
| KEL19-1 | 2880779 | 700360226 | 43.58 | 98.46 | 94.87 |
| KEL19-2 | 2791136 | 717128970 | 43.74 | 98.46 | 94.75 |
| KEL19-3 | 3419313 | 836230735 | 42.39 | 98.32 | 94.49 |
| KEL19-4 | 2757958 | 710051282 | 42.00 | 98.41 | 94.57 |
| KEL19-5 | 3107259 | 776637547 | 42.42 | 98.48 | 94.94 |
| KS19-1 | 3260016 | 819685505 | 43.46 | 98.42 | 94.53 |
| KS19-2 | 3536088 | 876915098 | 43.04 | 98.38 | 94.52 |
| KS19-3 | 3086903 | 769320407 | 43.20 | 98.35 | 94.36 |
| KS19-4 | 2859883 | 725740073 | 43.91 | 98.42 | 94.58 |
| KS19-5 | 3408877 | 862919215 | 44.68 | 98.41 | 94.53 |
| SC20-1 | 2124921 | 564938423 | 44.55 | 98.10 | 93.22 |
| SC20-2 | 2405673 | 658038358 | 44.57 | 98.11 | 93.02 |
| SC20-3 | 2887975 | 749758996 | 43.86 | 98.18 | 93.45 |
| SC20-4 | 2720863 | 710290248 | 46.54 | 98.23 | 93.51 |
| SC20-5 | 2315748 | 617679274 | 43.49 | 98.11 | 93.25 |
| SW20-1 | 3648140 | 928855019 | 44.33 | 98.31 | 94.15 |
| SW20-2 | 3187571 | 823767737 | 44.17 | 98.28 | 93.98 |
| SW20-3 | 3459551 | 894270578 | 44.65 | 98.34 | 94.16 |
| SW20-4 | 3597835 | 906447236 | 44.14 | 98.35 | 94.33 |
| Mean | 2893829.59 | 732442384.10 | 43.77 | 98.35 | 94.30 |
| Sum | 141797650 | 35889676823 | - | - | - |

Notes：

Reads：Number of clean PE reads per sample
Bases：Number of clean bases per sample

GC：GC content of each sample

Q20：Percentage of bases with mass number greater than 20
Q30：Percentage of bases with mass number greater than 30
