# Supplementary material for: Genetic diversity and population structure of the natural population of Helicoverpa armigera in Northwest China using Genotyping by Sequencing (GBS) technology
Source: PLoS One. 2025 Nov 6;20(11):e0336253. doi: 10.1371/journal.pone.0336253 (PMC12591424; doi:10.1371/journal.pone.0336253)
Supplement: S2 Table — (DOCX) [file pone.0336253.s002.docx]

**Table S2 Comparison rate of sequencing data**

| ID | Total reads | No.uniquely mapped | %uniquely mapped | No.repeatly mapped | % repeatly mapped | No.unmapped | % unmapped |
| --- | --- | --- | --- | --- | --- | --- | --- |
| AKS19-1 | 2907723 | 2153584 | 74.06% | 232892 | 8.01% | 521247 | 17.93% |
| AKS19-2 | 2897811 | 2244483 | 77.45% | 270850 | 9.35% | 382478 | 13.20% |
| AKS19-3 | 3078904 | 2207990 | 71.71% | 254085 | 8.25% | 616829 | 20.03% |
| AKS19-4 | 2396320 | 1877701 | 78.36% | 231587 | 9.66% | 287032 | 11.98% |
| AKS19-5 | 3162823 | 1797821 | 56.84% | 213155 | 6.74% | 1151847 | 36.42% |
| AKS20-1 | 2010151 | 207100 | 10.30% | 38590 | 1.92% | 1764461 | 87.78% |
| AKS20-2 | 3557576 | 2451544 | 68.91% | 288120 | 8.10% | 817912 | 22.99% |
| AKS20-3 | 2035939 | 1034938 | 50.83% | 108785 | 5.34% | 892216 | 43.82% |
| AKS20-4 | 3288536 | 2396920 | 72.89% | 296080 | 9.00% | 595536 | 18.11% |
| AKS20-5 | 2678466 | 1139108 | 42.53% | 129789 | 4.85% | 1409569 | 52.63% |
| ALE19-1 | 3474584 | 2647997 | 76.21% | 326747 | 9.40% | 499840 | 14.39% |
| ALE19-2 | 3164167 | 2237627 | 70.72% | 283307 | 8.95% | 643233 | 20.33% |
| ALE19-3 | 2796803 | 1194454 | 42.71% | 144594 | 5.17% | 1457755 | 52.12% |
| ALE19-4 | 3065895 | 2023844 | 66.01% | 228621 | 7.46% | 813430 | 26.53% |
| ALE19-5 | 2586490 | 1745488 | 67.48% | 151748 | 5.87% | 689254 | 26.65% |
| CJ19-1 | 2710358 | 2134740 | 78.76% | 261685 | 9.65% | 313933 | 11.58% |
| CJ19-2 | 2785730 | 2190067 | 78.62% | 274695 | 9.86% | 320968 | 11.52% |
| CJ19-3 | 3162006 | 2469087 | 78.09% | 316939 | 10.02% | 375980 | 11.89% |
| CJ19-4 | 2125493 | 1432315 | 67.39% | 146456 | 6.89% | 546722 | 25.72% |
| CJ19-5 | 2219606 | 1730476 | 77.96% | 211083 | 9.51% | 278047 | 12.53% |
| CJ20-1 | 1552089 | 1210789 | 78.01% | 159254 | 10.26% | 182046 | 11.73% |
| CJ20-2 | 3150473 | 2451389 | 77.81% | 298850 | 9.49% | 400234 | 12.70% |
| CJ20-3 | 2968838 | 2219506 | 74.76% | 289665 | 9.76% | 459667 | 15.48% |
| CJ20-4 | 2244501 | 1682781 | 74.97% | 186857 | 8.33% | 374863 | 16.70% |
| CJ20-5 | 2612318 | 1839009 | 70.40% | 188137 | 7.20% | 585172 | 22.40% |
| HM19-1 | 2964083 | 2102240 | 70.92% | 231501 | 7.81% | 630342 | 21.27% |
| HM19-2 | 3698193 | 2762996 | 74.71% | 351561 | 9.51% | 583636 | 15.78% |
| HM19-3 | 3430109 | 2626219 | 76.56% | 357385 | 10.42% | 446505 | 13.02% |
| HM19-4 | 2387831 | 1725649 | 72.27% | 212431 | 8.90% | 449751 | 18.84% |
| HM19-5 | 3227345 | 2394321 | 74.19% | 297975 | 9.23% | 535049 | 16.58% |
| KEL19-1 | 2880779 | 2202375 | 76.45% | 294120 | 10.21% | 384284 | 13.34% |
| KEL19-2 | 2791136 | 2030691 | 72.76% | 220472 | 7.90% | 539973 | 19.35% |
| KEL19-3 | 3419313 | 2632593 | 76.99% | 325193 | 9.51% | 461527 | 13.50% |
| KEL19-4 | 2757958 | 1945081 | 70.53% | 215318 | 7.81% | 597559 | 21.67% |
| KEL19-5 | 3107259 | 2380152 | 76.60% | 278108 | 8.95% | 448999 | 14.45% |
| KS19-1 | 3260016 | 2314317 | 70.99% | 291879 | 8.95% | 653820 | 20.06% |
| KS19-2 | 3536088 | 2669419 | 75.49% | 351609 | 9.94% | 515060 | 14.57% |
| KS19-3 | 3086903 | 2332529 | 75.56% | 298746 | 9.68% | 455628 | 14.76% |
| KS19-4 | 2859883 | 2090460 | 73.10% | 256041 | 8.95% | 513382 | 17.95% |
| KS19-5 | 3408877 | 2583096 | 75.78% | 316031 | 9.27% | 509750 | 14.95% |
| SC20-1 | 2124921 | 1539742 | 72.46% | 154718 | 7.28% | 430461 | 20.26% |
| SC20-2 | 2405673 | 1457410 | 60.58% | 101002 | 4.20% | 847261 | 35.22% |
| SC20-3 | 2887975 | 2192689 | 75.92% | 257194 | 8.91% | 438092 | 15.17% |
| SC20-4 | 2720863 | 1552000 | 57.04% | 182809 | 6.72% | 986054 | 36.24% |
| SC20-5 | 2315748 | 1589664 | 68.65% | 173558 | 7.49% | 552526 | 23.86% |
| SW20-1 | 3648140 | 2417543 | 66.27% | 403999 | 11.07% | 826598 | 22.66% |
| SW20-2 | 3187571 | 2006657 | 62.95% | 314636 | 9.87% | 866278 | 27.18% |
| SW20-3 | 3459551 | 2166634 | 62.63% | 353257 | 10.21% | 939660 | 27.16% |
| SW20-4 | 3597835 | 2140794 | 59.50% | 423130 | 11.76% | 1033911 | 28.74% |
| Mean | 2893830 | 2011756 | 69.03% | 248883 | 8.44% | 633191 | 22.53% |

Notes：

ID：Sample number

Total reads：The number of reads per sample

Uniquely mapped：Reads mapped to a unique location in the genome

Repeatedly mapped：Reads mapped to multiple locations in the genome

Unmapped：We can't compare the reads of the genome
