# Supplementary material for: Genetic diversity and population structure of the natural population of Helicoverpa armigera in Northwest China using Genotyping by Sequencing (GBS) technology
Source: PLoS One. 2025 Nov 6;20(11):e0336253. doi: 10.1371/journal.pone.0336253 (PMC12591424; doi:10.1371/journal.pone.0336253)
Supplement: S3 Table — (DOCX) [file pone.0336253.s003.docx]

**Table S3 Statistical table of cotton bollworm genome coverage and coverage depth**

| Sample | Coverage(%) | Mean Depth |
| --- | --- | --- |
| AKS19-1 | 4.23 | 34.17 |
| AKS19-2 | 4.38 | 32.96 |
| AKS19-3 | 5.06 | 27.80 |
| AKS19-4 | 3.68 | 32.61 |
| AKS19-5 | 5.13 | 23.18 |
| AKS20-1 | 1.50 | 3.73 |
| AKS20-2 | 9.84 | 15.61 |
| AKS20-3 | 5.26 | 13.08 |
| AKS20-4 | 8.34 | 17.92 |
| AKS20-5 | 5.12 | 14.68 |
| ALE19-1 | 6.14 | 26.89 |
| ALE19-2 | 5.50 | 25.28 |
| ALE19-3 | 4.42 | 16.69 |
| ALE19-4 | 5.93 | 21.72 |
| ALE19-5 | 5.41 | 22.35 |
| CJ19-1 | 5.08 | 25.52 |
| CJ19-2 | 4.99 | 25.31 |
| CJ19-3 | 5.74 | 25.07 |
| CJ19-4 | 5.61 | 16.75 |
| CJ19-5 | 4.75 | 22.04 |
| CJ20-1 | 3.34 | 21.38 |
| CJ20-2 | 5.46 | 27.32 |
| CJ20-3 | 5.63 | 22.69 |
| CJ20-4 | 4.89 | 20.86 |
| CJ20-5 | 7.27 | 16.71 |
| HM19-1 | 5.71 | 23.67 |
| HM19-2 | 5.46 | 30.13 |
| HM19-3 | 6.02 | 25.25 |
| HM19-4 | 6.08 | 17.76 |
| HM19-5 | 5.51 | 26.99 |
| KEL19-1 | 5.47 | 23.65 |
| KEL19-2 | 4.03 | 33.72 |
| KEL19-3 | 7.24 | 21.68 |
| KEL19-4 | 6.49 | 18.90 |
| KEL19-5 | 6.04 | 24.14 |
| KS19-1 | 6.00 | 24.35 |
| KS19-2 | 6.13 | 26.51 |
| KS19-3 | 5.62 | 25.12 |
| KS19-4 | 5.62 | 23.58 |
| KS19-5 | 4.66 | 33.81 |
| SC20-1 | 4.54 | 23.59 |
| SC20-2 | 5.93 | 18.22 |
| SC20-3 | 5.32 | 27.53 |
| SC20-4 | 4.59 | 22.12 |
| SC20-5 | 5.10 | 21.75 |
| SW20-1 | 3.79 | 37.48 |
| SW20-2 | 4.50 | 26.94 |
| SW20-3 | 4.05 | 33.04 |
| SW20-4 | 5.30 | 23.16 |
| Mean | 5.34 | 23.78 |

Notes：

Coverage：Proportion of sequencing data covering the whole genome

Mean Depth：Average coverage depth
