# Supplementary material for: Genetic diversity and population structure of the natural population of Helicoverpa armigera in Northwest China using Genotyping by Sequencing (GBS) technology
Source: PLoS One. 2025 Nov 6;20(11):e0336253. doi: 10.1371/journal.pone.0336253 (PMC12591424; doi:10.1371/journal.pone.0336253)
Supplement: S4 Table — (DOCX) [file pone.0336253.s004.docx]

**Table S4 ANNOVAR annotation classification**

| Category | Explanation |
| --- | --- |
| exonic | variant overlaps a coding |
| splicing | variant is within 2-bp of a splicing junction |
| ncRNA | variant overlaps a transcript without coding annotation in the gene definition |
| UTR5 | variant overlaps a 5’ untranslated region |
| UTR3 | variant overlaps a 3’ untranslatedregion |
| intronic | variant overlaps an intron |
| upstream | variant overlaps 1-kb region upstream of transcription start site |
| downstream | variant overlaps 1-kb region downtream of transcription end site |
| intergenic | variant is in intergenic region |
