# Supplementary material for: Genetic diversity and population structure of the natural population of Helicoverpa armigera in Northwest China using Genotyping by Sequencing (GBS) technology
Source: PLoS One. 2025 Nov 6;20(11):e0336253. doi: 10.1371/journal.pone.0336253 (PMC12591424; doi:10.1371/journal.pone.0336253)
Supplement: S5 Table — (DOCX) [file pone.0336253.s005.docx]

**Table S5 Summary of SNP annotation results**

| Category | Number | Ratio |
| --- | --- | --- |
| intergenic | 15803 | 22.51% |
| upstream/downstream | 2652 | 3.78% |
| upstream | 1306 | 1.86% |
| downstream | 1074 | 1.53% |
| upstream&downstream | 272 | 0.39% |
| genic | 51743 | 73.71% |
| intronic | 26269 | 37.42% |
| exonic | 23273 | 33.15% |
| synonymous | 21690 | 30.90% |
| nonsynonymous | 1570 | 2.24% |
| stopgain | 12 | 0.02% |
| stoploss | 1 | 0.00% |
| UTR5 | 484 | 0.69% |
| UTR3 | 1150 | 1.64% |
| splicing | 6 | 0.01% |
