# Supplementary material for: Genetic diversity and population structure of the natural population of Helicoverpa armigera in Northwest China using Genotyping by Sequencing (GBS) technology
Source: PLoS One. 2025 Nov 6;20(11):e0336253. doi: 10.1371/journal.pone.0336253 (PMC12591424; doi:10.1371/journal.pone.0336253)
Supplement: S6 Table — (DOCX) [file pone.0336253.s006.docx]

**Table S6 Summary of InDel annotation results**

| Category | Number | Ratio |
| --- | --- | --- |
| intergenic | 2237 | 31.44% |
| upstream/downstream | 428 | 6.01% |
| upstream | 206 | 2.89% |
| downstream | 181 | 2.54% |
| upstream&downstream | 41 | 0.58% |
| genic | 4451 | 62.55% |
| intronic | 4020 | 56.49% |
| exonic | 155 | 2.18% |
| synonymous | 41 | 0.58% |
| nonsynonymous | 111 | 1.56% |
| stopgain | 3 | 0.04% |
| stoploss | 0 | 0.00% |
| UTR5 | 63 | 0.89% |
| UTR3 | 195 | 2.74% |
| splicing | 14 | 0.20% |
