# Supplementary material for: Genetic diversity and population structure of the natural population of Helicoverpa armigera in Northwest China using Genotyping by Sequencing (GBS) technology
Source: PLoS One. 2025 Nov 6;20(11):e0336253. doi: 10.1371/journal.pone.0336253 (PMC12591424; doi:10.1371/journal.pone.0336253)
Supplement: S7 Table — (DOCX) [file pone.0336253.s007.docx]

**Table S7 Statistical table of variation quantity and density of SNP and InDel markers**

| Chr | Length | No. SNPs | SNP density | No. InDels | InDel density |
| --- | --- | --- | --- | --- | --- |
| 1 | 6146627 | 1,707 | 277.71 | 206 | 33.51 |
| 2 | 430152 | 733 | 170.40 | 92 | 21.39 |
| 3 | 3826781 | 747 | 195.20 | 116 | 30.31 |
| 4 | 3891429 | 915 | 235.13 | 78 | 20.04 |
| 5 | 3592362 | 928 | 258.33 | 84 | 23.38 |
| 6 | 3068546 | 551 | 179.56 | 58 | 18.90 |
| 7 | 3107902 | 713 | 229.42 | 73 | 23.49 |
| 8 | 3096529 | 1036 | 334.57 | 105 | 33.91 |
| 9 | 3005218 | 515 | 171.37 | 32 | 10.65 |
| 10 | 3053552 | 840 | 275.09 | 75 | 24.56 |
| 11 | 2905865 | 609 | 209.58 | 70 | 24.09 |
| 12 | 2918994 | 469 | 160.67 | 44 | 15.07 |
| 13 | 2877172 | 549 | 190.81 | 71 | 24.68 |
| 14 | 2788665 | 686 | 246.00 | 95 | 34.07 |
| 15 | 2699263 | 380 | 140.78 | 28 | 10.37 |
| Whole | 51280425 | 11378 | 221.88 | 1227 | 23.93 |
